# Refuges of conventional host plants counter dominant resistance of cotton bollworm to transgenic Bt cotton

**DOI:** 10.1016/j.isci.2023.106768

**Published:** 2023-04-26

**Authors:** Fang Guan, Xiaoguang Dai, Bofeng Hou, Shuwen Wu, Yihua Yang, Yanhui Lu, Kongming Wu, Bruce E. Tabashnik, Yidong Wu

**Affiliations:** 1 College of Plant Protection, Nanjing Agricultural University, Nanjing, China; 2 Institute of Plant Protection, Chinese Academy of Agricultural Sciences, Beijing, China; 3 Department of Entomology, University of Arizona, Tucson, AZ 85721, USA

**Keywords:** Evolutionary ecology, Sequence analysis, Interaction of plants with organisms

## Abstract

Transgenic crops have revolutionized insect pest control, but evolution of resistance by pests threatens their continued success. The primary strategy for combating pest resistance to crops producing insecticidal proteins from *Bacillus thuringiensis* (Bt) uses refuges of non-Bt host plants to allow survival of susceptible insects. The prevailing paradigm is that refuges delay resistance that is rare and recessively inherited. However, we discovered refuges countered resistance to Bt cotton that was neither rare nor recessive. In a 15-year field study of the cotton bollworm, the frequency of a mutation conferring dominant resistance to Bt cotton surged 100-fold from 2006 to 2016 yet did not rise from 2016 to 2020. Computer simulations indicate the increased refuge percentage from 2016 to 2020 is sufficient to explain the observed halt in the evolution of resistance. The results also demonstrate the efficacy of a Bt crop can be sustained by non-Bt refuges of other crops.

## Highlights

• Evolution of resistance by pests threatens the continued success of Bt crops
• Refuges can delay evolution of Bt resistance that is neither rare nor recessive
• The efficacy of a Bt crop can be sustained by non-Bt refuges of other crops

## Graphical abstract

**Figure:**
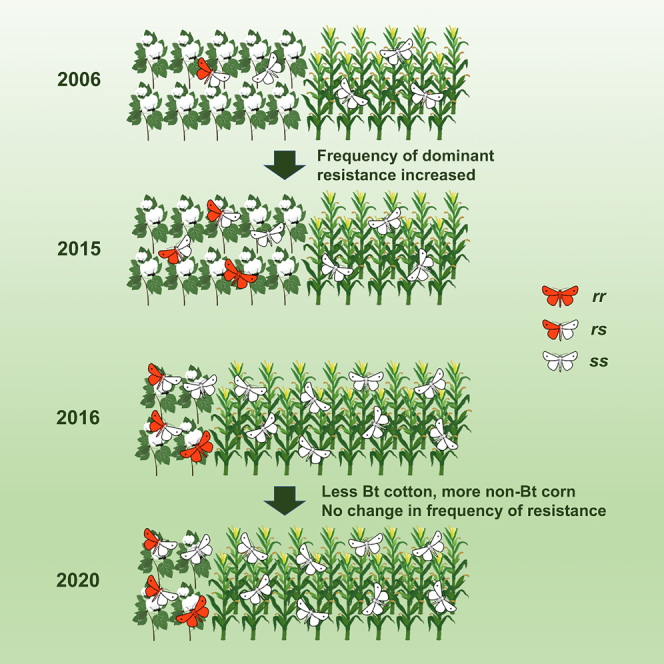

## Introduction

From 1996 to 2019, farmers planted a total of over 1 billion hectares (ha) of genetically engineered crops that produce insecticidal proteins from the bacterium *Bacillus thuringiensis* (Bt).^1^ These transgenic Bt crops can suppress pests, reduce insecticide sprays, enhance biological control, and increase farmer profits.^2^^,^^3^^,^^4^^,^^5^^,^^6^^,^^7^^,^^8^ However, evolution of pest resistance to Bt crops has diminished these benefits.^9^ The number of cases of practical resistance to Bt crops, which is field-evolved resistance that decreases the efficacy of the Bt crop and has practical consequences for pest control, surged from 3 in 2005 to 26 by 2020.^10^^,^^11^^,^^12^ Thus, better understanding of factors affecting evolution of pest resistance to Bt crops is urgently needed to sustain the efficacy of this technology.

The primary strategy for delaying pest resistance to Bt crops entails refuges of non-Bt host plants that enable survival of susceptible pests to mate with resistant pests.^9^^,^^13^^,^^14^ Refuges can include non-Bt varieties of the same plant species as the Bt crop, as well as non-Bt plants of other species, which are sometimes called “natural refuges”.^15^ Refuges are especially effective for delaying resistance that is rare and inherited as a recessive trait, because the matings between extremely rare homozygous resistant insects and relatively abundant homozygous susceptible insects from refuges produce heterozygous progeny that are killed by the Bt crop.^9^^,^^14^

Some modeling results and empirical observations suggest that sufficiently large refuges can also delay evolution of resistance that is either not rare or not recessive,^12^^,^^14^^,^^15^^,^^16^ but we are not aware of previous work rigorously testing the ability of refuges to delay resistance to a single toxin that is neither rare nor recessive. In this context, resistance is usually considered rare if the resistance allele frequency is not greater than 0.001, and recessive if the dominance parameter *h* (which ranges from 0 for completely recessive to 1 for completely dominant) is close to zero.^9^^,^^14^

Here, we tested the hypothesis that refuges can delay evolution of resistance that is not rare or recessive by comparing predictions from computer simulations with field monitoring data from a 15-year study of the cotton bollworm, *Helicoverpa armigera*. This mobile, polyphagous lepidopteran is one of the world’s most damaging pests, causing over $3 billion in estimated losses annually.^17^^,^^18^ It is widely distributed throughout Asia, Africa, Australia, and Europe and recently invaded the Americas.^17^^,^^18^

## Results

### Monitoring resistance with DNA screening and bioassays

We assessed *H. armigera* from six provinces of northern China, where transgenic cotton producing Bt toxin Cry1Ac has been planted by millions of smallholder farmers since 1997.^15^^,^^19^ We analyzed results from 140,362 insects derived from 25 field sites during 2006–2020, including screening DNA of 10,954 field-collected moths, bioassays determining resistance to Cry1Ac of 129,408 larvae, and evaluation of 1370 individuals by bioassays and DNA screening (Figure S1).

Previous work showed that a single base pair substitution (T92C) in a tetraspanin gene (*HaTSPAN1*) confers dominant resistance of *H. armigera* to Cry1Ac (*h* = 0.56 to 1.0).^19^ Genome-wide association studies of 21 field-derived backcross families indicated this mutation is the primary genetic basis of nonrecessive *H. armigera* resistance to Cry1Ac in northern China.^20^ In addition, DNA screening showed that the frequency of this mutation rose in northern China from 0.001 in 2006 to 0.10 in 2016.^19^ Also, previous results from bioassays testing field-derived larvae at a diagnostic concentration of Cry1Ac (1 μg Cry1Ac per cm^2^ diet) showed that the percentage of individuals resistant to Cry1Ac increased significantly in this region, from 0.93% in 2010 to 5.5% in 2013.^15^

**Figure 1:**
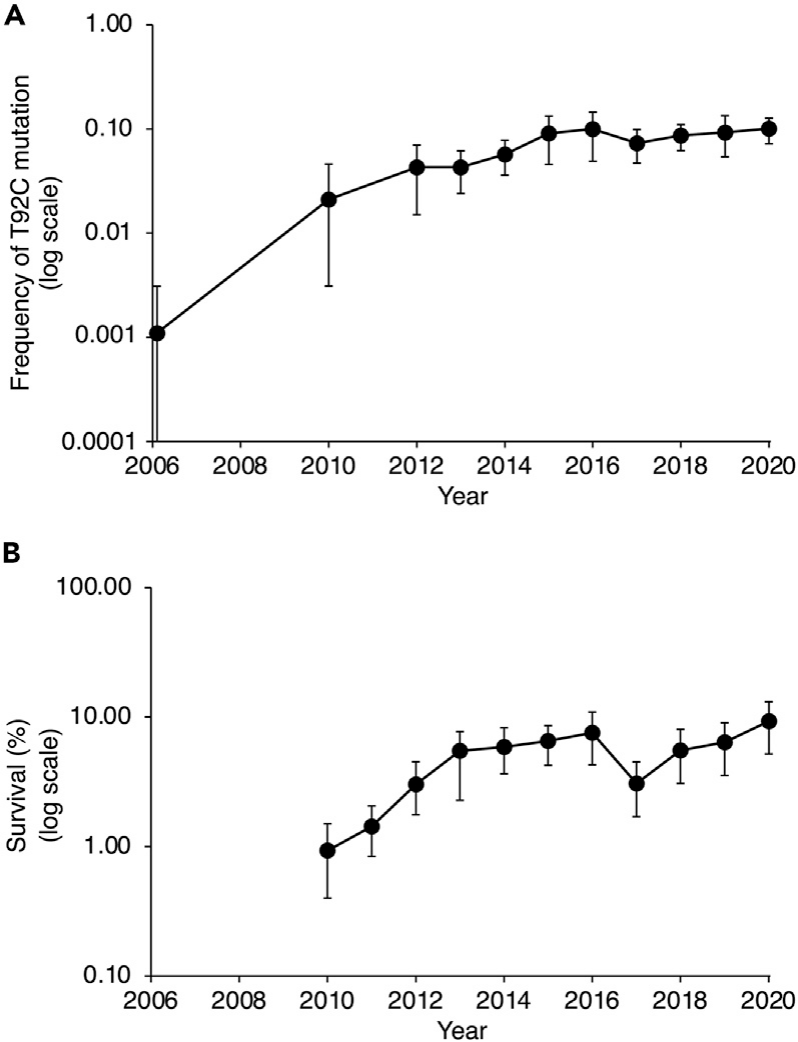
Monitoring resistance of *H. armigera* to Bt toxin Cry1Ac in northern China. Values are means and 95% CIs (A) Frequency of the T92C mutation based on DNA screening of pooled moths (average of 996 moths screened per year, range: 469 to 2050; mean: 8.4 sites per year, range: 6 to 13). The lower 95% limit for 2006 is zero, which cannot be plotted on a log scale. (B) Survival (%) of the F_1_ progeny of field-collected larvae tested at the diagnostic concentration of Cry1Ac (average of 11,764 field-derived larvae tested per year, range: 7536 to 15,480). Data were reported previously for 2006 to 2016 for (A)^19^ and 2010 to 2013 for (B).^15^

Here we report new bioassay data from northern China showing the percentage of larvae resistant to Cry1Ac reached 7.6% in 2016 (95% CI: 4.3–10.9%) and increased significantly from 2010 to 2016 (linear regression of log-transformed data: *R*^*2*^ = 0.88, log (y) = 0.16x – 312, df = 5, p = 0.002; Figure 1). Based on these new bioassay results and the previously reported data from bioassays and DNA screening, rapid increases in resistance to Cry1Ac were expected after 2016. In particular, resistance conferred by the T92C mutation was expected to rise quickly because this mutation is not recessive and its frequency was 0.10 in 2016^19^–100 times greater than the standard threshold for rarity.^9^^,^^14^

Contrary to this expectation, we discovered that resistance to Cry1Ac did not increase significantly from 2016 to 2020 (Figure 1). In striking contrast with the 100-fold increase in the frequency of the T92C mutation from 2006 to 2016, this frequency was 0.10 in 2016 and 2020 (95% CI, 2016: 0.05–0.14; 2020: 0.08–0.13; Figure 1A, Table S1). Also, unlike the 8-fold increase in the percentage of larvae resistant to Cry1Ac in bioassays from 2010 to 2016, this percentage did not increase significantly from 2016 (mean: 7.6%) to 2020 (mean 9.3%, 95% CI: 5.2–13.1%; linear regression of log-transformed data: *R*^2^ = 0.18, log (y) = 0.05x – 98, df = 3, p = 0.47; Figure 1B, Table S2).

We obtained data from both monitoring methods (bioassays and DNA screening) in each of 10 years: 2010 and 2012–2020 (Figure 1). Across these 10 years, the percentage of resistant larvae in bioassays was correlated with frequency of the T92C mutation (log-transformed data: *r* = 0.84, df = 8, p = 0.002, Figure S2). These results imply that the frequency of the T92C mutation is a reasonable indicator of the overall percentage of resistant larvae from 2010 to 2020.

**Figure 2:**
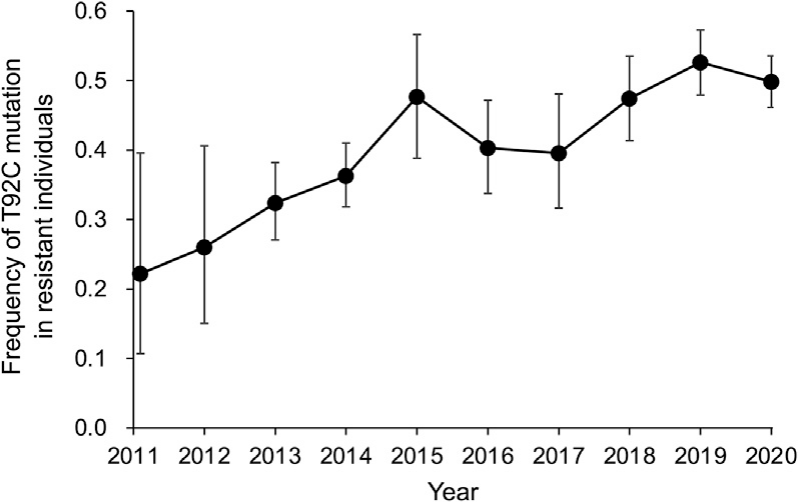
The frequency of the T92C mutation in resistant, field-derived *H. armigera* from northern China Based on screening DNA individually from an average of 137 resistant F_1_ moths per year (range: 18 to 360, Table S3). Bars show 95% CI. Linear regression indicates a significant increase from 2011 to 2016 (slope = 0.046, *R*^2^ = 0.83, df = 4, p = 0.012) and from 2016 to 2020 (slope = 0.032 *R*^2^ = 0.77, df = 3, p = 0.0497). Data were reported previously for 2011 to 2016.^19^

To more directly evaluate the importance of the T92C mutation in field-evolved resistance, we screened the DNA of insects that were the first-generation (F_1_) progeny of field-collected insects and were phenotypically resistant based on their survival in bioassays at the diagnostic concentration of Cry1Ac. From 2016 to 2020, an annual mean of 79% (SE: 3%) of the phenotypically resistant individuals carried at least one copy of the T92C mutation, indicating this dominant mutation was a primary contributor to resistance during this 5-year period (Figure 2, Table S3). The contributions to resistance of other mutations remain to be assessed.

As reported previously, the percentage of phenotypically resistant individuals carrying the T92C mutation increased significantly from 44% in 2011 to 70% in 2016.^19^ Here, we report that the frequency of the T92C mutation also increased significantly from 0.22 in 2011 to 0.40 in 2016 and from 0.40 in 2016 to 0.50 in 2020 (Figure 2, Table S3). These results support the general prediction from resistance management theory that has been confirmed by field outcomes elsewhere: dominantly inherited resistance evolves more readily than recessively inherited resistance.^9^^,^^14^^,^^15^^,^^16^^,^^19^

### Increase in the percentage of non-Bt host plants

**Figure 3:**
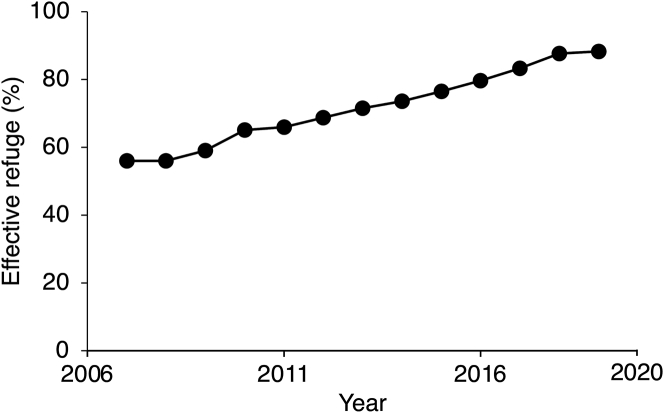
The effective refuge percentage, which incorporates non-Bt cotton and other non-Bt host plants of *H. armigera,* increased in northern China from 2007 to 2019 Linear regression: y = 2.85x – 5667, df = 11, *R*^2^ = 0.99, p < 0.0001.

To understand why resistance to Cry1Ac and the frequency of the T92C mutation increased significantly from 2010 to 2016, but not from 2016 to 2020, we investigated the possibility that this change in evolutionary trajectory was associated with an increase in the percentage of *H. armigera* host plants consisting of conventional non-Bt plants that acted as refuges. For the six provinces of northern China where we monitored *H. armigera* resistance to Cry1Ac, we calculated the “effective refuge percentage” for 2010 to 2019 based on the hectares of non-Bt cotton and other non-Bt host plants of *H. armigera* adjusted for their production of *H. armigera* relative to non-Bt cotton.^15^^,^^19^ This analysis revealed that the annual effective refuge percentage increased significantly from 2010 to 2019, with a mean of 66% from 2007 to 2015 versus 85% from 2016 to 2019 (t-test, *t* = 4.7, df = 11, p = 0.0007; Figure 3, Table S4). This reflects a decrease in the planting of cotton from 2.9 million ha in 2010 to 0.64 million ha in 2019 (a 78% decline), while the total hectares of *H. armigera* host plants were relatively constant at 27.7 and 27.2 million ha in 2010 and 2019, respectively (Table S4).

### Comparing predictions from computer simulations with the observed outcome

We used computer simulation modeling to determine if the observed increase in the effective refuge percentage was sufficient to explain the observed decrease in the rate of evolution of resistance to Cry1Ac. We tested three hypotheses about how refuges affected the evolution of resistance to Cry1Ac conferred by the dominant T92C mutation: i) refuges had no effect, ii) only non-Bt cotton refuges delayed resistance evolution, and iii) non-Bt cotton and other non-Bt host plants that contribute to the effective refuge percentage delayed resistance.

We simulated the period from 2006 to 2020 using a one-locus, two-allele population genetic model, with key parameters based on empirical data, including previously measured fitness values for each genotype on Bt and non-Bt cotton plants for individuals with zero, one, or two copies of the T92C mutation^15^^,^^19^ (Table S5). These empirically estimated parameters include the dominance of resistance to Bt cotton conferred by the T92C mutation (*h* = 0.79), a recessive fitness cost (0.36), and incomplete resistance (0.49).^19^ The recessive fitness cost reflects the finding that on non-Bt host plants, relative to a susceptible strain (SCD), fitness was lower for resistant homozygotes but not for heterozygotes.^19^ The incomplete resistance is based on the results showing fitness for the resistant strain was lower on Bt cotton than on non-Bt cotton.^19^

**Figure 4:**
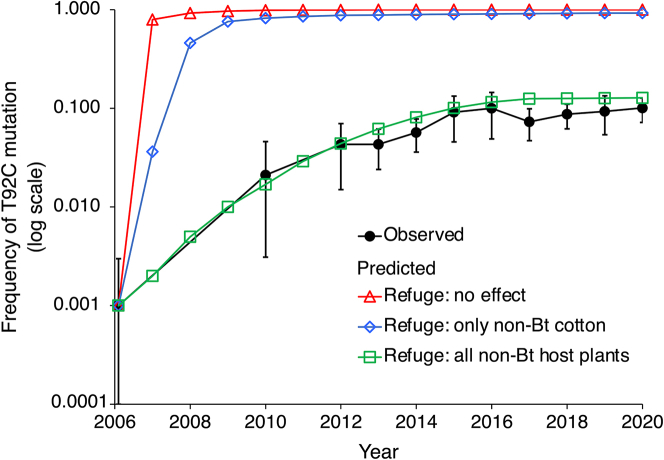
Observed versus predicted frequency of the *H. armigera* T92C mutation in northern China The observed values are means (with 95% CIs) based on DNA screening of pooled moths (data from Figure 1A). The predicted values are from simulations of a population genetic model with three different assumptions about refuges: i) refuges had no effect, ii) only refuges of non-Bt cotton plants delayed resistance, and iii) non-Bt cotton and other non-Bt host plants that contribute to the effective refuge percentage delayed resistance. The observed frequency for 2006 (mean: 0.001, 95% CI: 0.0–0.003) was used as the frequency for 2006 in all simulations (see supplemental information Appendix, Table S5).

Under the assumptions that refuges had no effect or that only non-Bt cotton plants acted as refuges, the T92C mutation frequency exceeded 0.75 by 2007 and 2009, respectively (Figure 4). Both of these projected rates of evolution of resistance are much faster than the observed trajectory of the T92C mutation (Figure 4). Thus, the simulation results do not support the hypotheses that refuges had no effect or that refuges of non-Bt cotton alone were sufficient to delay resistance. By contrast, under the assumption that non-Bt cotton and other non-Bt host plants acted as refuges that delayed resistance, the predicted trajectory corresponds well with the observed trajectory (Figure 4). This correspondence includes a predicted frequency of T92C in 2020 of 0.13 versus the observed frequency of 0.10 (Figure 4). Therefore, the simulation results indicate that together with the observed fitness cost and incomplete resistance, refuges of non-Bt cotton and other non-Bt host plants combined were sufficient to cause the observed delay in evolution of resistance to Cry1Ac conferred by the dominant T92C mutation. Additional sensitivity analyses show that the correspondence between predicted and observed outcomes is robust across a reasonable range of assumptions (Table S5, Figure S3).

### Potential effects of insecticide sprays on the change in resistance trajectory

We analyzed data on conventional insecticide treatments to test the alternative hypothesis that they could account for the change in the trajectory of resistance observed in northern China. We first tested the hypothesis that a decrease in sprays targeting *H. armigera* on non-cotton host plants increased the effective refuge percentage and thereby contributed to the halt in the evolution of resistance observed starting in 2016. However, the mean number of yearly sprays per ha targeting *H. armigera* on non-cotton host plants was actually higher from 2016 to 2019 (0.32, SE = 0.01) than 2007 to 2015 (0.26, SE = 0.01; *t* = 2.7, df = 11, p = 0.02, Table S6), which is opposite to the prediction from this hypothesis. Also not consistent with the hypothesis, the mean number of sprays targeting all pests on non-cotton host plants was not lower from 2016 to 2019 (2.0, SE = 0.2) than from 2007 to 2015 (1.7, SE = 0.04, *t* = 2.2, df = 11, p = 0.23, Table S7).

We also tested the related hypothesis that increased sprays targeting *H. armigera* on cotton, which was predominantly Bt cotton, increased the effective refuge percentage by decreasing the number of moths emerging from Bt cotton. Contradicting this hypothesis, the mean number of yearly sprays per ha targeting *H. armigera* on cotton was actually lower from 2016 to 2019 (1.6, SE = 0.08) than 2007 to 2015 (2.2, SE = 0.03; *t* = 8.0, df = 11, p < 0.001, Table S6). Also not supporting the hypothesis, the mean number of sprays targeting all pests on cotton was not higher from 2016 to 2019 (7.0, SE = 0.3) than from 2007 to 2015 (7.5, SE = 0.2, *t* = 1.7, df = 11, p = 0.13, Table S7). Thus, the data do not support the hypotheses that either decreases in sprays on non-cotton host plants or increases in sprays on cotton contributed to the observed change in the trajectory of resistance.

## Discussion

Resistance of *H. armigera* to Cry1Ac produced by Bt cotton had increased substantially in northern China by 2016 and was expected to escalate quickly thereafter.^15^^,^^19^ However, the results here show no significant increase occurred from 2016 to 2020 in either the frequency of the T92C mutation that confers dominant resistance to Cry1Ac or in the percentage of resistant larvae detected in bioassays. We also found that the mean annual effective refuge percentage, which includes contributions by non-Bt cotton and other non-Bt host plants, grew from 66% during 2007–2015 to 85% during 2016–2019. Our simulation modeling results indicate the observed increase in the effective refuge percentage was sufficient to cause the observed delay in evolution of resistance that occurred after 2016. Previous studies have provided modeling results and data indicating refuges can delay evolution of resistance to a single toxin that is recessive, rare, or both.^9^^,^^12^^,^^13^^,^^14^^,^^15^^,^^16^^,^^21^^,^^22^ However, we are not aware of previous empirical evidence demonstrating that refuges can also delay evolution of resistance to a single toxin that is neither rare nor recessive.

The observed increase in the effective refuge percentage in northern China reflects the 78% decrease in the planting of cotton from 2010 to 2019, and the concomitant increase in other host plants of *H. armigera*. Millions of smallholder farmers in northern China have reduced planting of cotton and increased planting of non-Bt corn and other non-Bt host plants of *H. armigera*, not as a resistance management strategy, but apparently based on economic considerations. This shift has been attributed to the increasing labor cost of cotton relative to other crops, decreased cotton price, and agricultural policies promoting cultivation of corn.^23^^,^^24^ Based on their study of three provinces (Hebei, Henan, and Shandong) representing half of the six provinces of northern China analyzed here, Lu et al.^25^ emphasized the drawbacks of this shift, including increases in moth abundance, yield loss, and insecticide sprays for host plants of *H. armigera* other than cotton.

Cultivation in northern China of corn, an *H. armigera* host plant that overlaps temporally with cotton,^26^ increased from 10.6 million ha in 2010 to 13.5 million ha in 2019 (Table S4). Thus, the ratio of corn to cotton ha rose from 3.7 in 2010 to 21 in 2019. Research in Hubei, one of the provinces studied here, showed the abundance of *H. armigera* in cotton was positively associated with the area planted to corn.^27^ This result is consistent with the idea that corn acted as a refuge for Bt cotton. Also supporting this hypothesis, a study in northern China using carbon isotope analysis revealed that 40 to 57% of *H. armigera* moths trapped in Bt cotton fields fed as larvae on C_4_ plants such as corn, rather than C_3_ host plants such as cotton.^28^ These results with *H. armigera* in northern China parallel analogous work with the closely related species *Helicoverpa zea* in the southern United States, where a substantial proportion of moths captured in cotton ate corn as larvae^29^^,^^30^ and damage to cotton was positively associated with the area planted to corn.^31^

As in northern China, *H. armigera* has not evolved practical resistance to Cry1Ac in Australia, despite its nonrecessive inheritance of resistance reported from there (*h* = 0.3)^32^ and its exposure to this toxin in Bt cotton for more than 20 years^33^ Unlike northern China where farmers have not switched to transgenic cotton producing two or more Bt toxins, growers in Australia planted single-toxin cotton producing Cry1Ac for only 8 years (1996–2003).^3^ During this period, the minimum refuge requirement was 70% non-Bt cotton and resistance to Cry1Ac remained rare.^3^ In 2004, Australian farmers switched completely to two-toxin Bt cotton producing Cry1Ac and Cry2Ab, then in 2016 to three-toxin Bt cotton producing Cry1Ac, Cry2Ab, and the Bt vegetative insecticidal protein Vip3Aa.^3^^,^^9^

In both China and Australia, cotton is the only Bt crop. So, all host plants other than cotton do not produce Bt toxins and have the potential to act as refuges for *H. armigera* and other cotton pests. Conversely, in the United States, where Bt cultivars accounted for a mean of over 75% of the hectares of corn, as well as cotton from 2009 to 2020,^34^ many populations of *H. zea* have evolved practical resistance to Cry1Ac and the other lepidopteran-active crystalline (Cry) toxins produced by Bt crops.^12^^,^^35^^,^^36^^,^^37^^,^^38^^,^^39^ In Brazil, where farmers have planted Bt corn, cotton, and soy to control fall armyworm (*Spodoptera frugipdera*), this polyphagous pest rapidly evolved resistance to the Cry toxins produced by these crops.^40^

The results here, and the other field outcomes summarized above suggest that refuges of non-Bt host plant species other than the Bt crop are likely to be essential for achieving effective refuge percentages high enough to substantially delay evolution of nonrecessive resistance to Bt crops in polyphagous pests. In particular, the potential for corn to delay evolution of resistance to Bt cotton by pests, such as *H. armigera* and *H. zea* has important implications for resistance management strategies in China, the United States, and elsewhere. In China, introduction of Bt corn is being considered to address the recent invasion of the fall armyworm.^41^^,^^42^^,^^43^ However, the results here suggest that widespread adoption of Bt corn in northern China could reduce the effective refuge percentage for *H. armigera*, thereby accelerating its evolution of resistance to both Bt cotton and Bt corn.

The dramatic delay in the evolution of *H. armigera* resistance to Cry1Ac in northern China from 2016 to 2020 could represent a genetic equilibrium^44^ where the strong selection for resistance on Bt cotton was balanced by the weaker selection against resistance mediated by fitness costs on the non-Bt host plants that accounted for a larger proportion of the pest’s habitat. Nonetheless, the results reported here do not preclude future increases in resistance. Evolution of resistance could be accelerated by reductions in the effective refuge percentage, as well as by emergence of new mutations that confer resistance that is more complete, more dominant, or has lower fitness costs. Accordingly, before resistance to Cry1Ac becomes a serious problem, we support introduction of transgenic cotton producing several Bt toxins (e.g., Cry1Ac, Cry2Ab, and Vip3Aa) to sustain efficacy against *H. armigera* and other pests.^22^^,^^45^

### Limitations of the study

Our simulation modeling results indicate the observed increase in the effective refuge percentage was sufficient to cause the observed delay in evolution of resistance that occurred after 2016. However, we cannot exclude the potential effects of other factors that we did not evaluate.

## STAR★Methods

### Key resources table

**Table:** 

REAGENT or RESOURCE	SOURCE	IDENTIFIER
**Software and algorithms**		

FastQC	Open source	http://www.bioinformatics.babraham.ac.uk/projects/fastqc
FASTX_Clipper	Open source	http://hannonlab.cshl.edu/fastx_toolkit
FASTX_Collapser	Open source	http://hannonlab.cshl.edu/fastx_toolkit
FASTQ_Quality_Filter	Open source	http://hannonlab.cshl.edu/fastx_toolkit
PANDAseq	Masella et al.^46^	https://github.com/neufeld/pandaseq
BWA MEM	Li^47^	https://bio-bwa.sourceforge.net
Python Script-1	Guan et al.^48^	https://onlinelibrary.wiley.com/doi/10.1002/ps.6192
Python Script-2	Guan et al.^48^	https://onlinelibrary.wiley.com/doi/10.1002/ps.6192
Seqtk_demultiplex	Open source	https://github.com/jameslz/fastx-utils

**Deposited data**		

Amplicon sequencing data	This paper	BioProject No. PRJNA919153

### Resource availability

#### Lead contact

Any requests for resources and information about methodology should be directed to and will be fulfilled by lead contact Yidong Wu (wyd@njau.edu.cn).

#### Materials availability

This study did not generate new unique reagents.

### Experimental model and subject details

We collected male and female cotton bollworm (*Helicoverpa armigera*) moths from cotton fields with light traps (250 W) at 21 sites in northern China (Figure S1) during June to August of 2006, 2010, and 2012–2020 and screened their DNA for the T92C mutation using previously described methods.^19^ The moths were preserved in 100% ethanol at room temperature and allowed to air dry for 10 min to evaporate the ethanol before extracting DNA. We screened DNA from field-collected moths for the T92C mutation using pooled legs as detailed below. Previous results from 2010-2016 showed that the DNA screening results from pooled moths and individual moths were highly correlated (*r* = 0.98).^19^

### Methods details

#### Bioassays to measure the percentage of resistant individuals in field populations

We collected *H. armigera* from cotton fields at 17 sites in northern China (Figure S1) from June to August 2010–2020 and tested their F_1_ progeny in bioassays using previously described methods.^15^

We collected male and female moths by light trap at 13 of the 17 field sites in northern China from 2010-2020. We collected eggs on Bt cotton at the other four field sites in northern China (Anqing, Jingzhou, Qianjiang and Yancheng) from 2010-2020. We collected 29,210 *H. armigera* from the field in 123 samples (mean = 237 individuals per sample). We collected 14,796 moths in 93 samples (mean = 159 moths per sample) and 14,414 eggs in 30 samples (mean = 480 eggs per sample). To increase representation of the diversity within field populations, we collected only one egg per cotton plant.

Larvae from field-collected eggs and from eggs obtained in the lab from field-collected moths were reared to adults in the laboratory on untreated artificial diet.^49^ From each field site, we pooled adults for mass mating to produce F_1_ progeny. We used diet bioassays^49^ to test a mean of 1052 F_1_ larvae per site per year at the diagnostic concentration of Cry1Ac (1 microgram Cry1Ac per cm^2^ diet; Table S2). Larvae from 123 strains derived from the field during 2010–2020 (Table S2) were reared on artificial diet and adults were maintained as described previously.^49^

In addition to testing 129,408 F_1_ progeny of field-collected insects from 17 sites in northern China, we tested 168 to 1008 larvae of the susceptible SCD lab strain of *H. armigera* each year from 2010 to 2020 as a control (total = 8808 SCD larvae, mean = 800 SCD larvae per year). SCD was started with insects from the Côte D’Ivoire (Ivory Coast), Africa over 30 years ago and was maintained in the laboratory without exposure to insecticides or Bt toxins.^19^ For SCD larvae, mortality was 100% at the diagnostic concentration of Cry1Ac and 1.5% on control diet that was not treated with Cry1Ac (range: 0 to 6.3%).

#### Amplicon sequencing of pooled moth legs to detect T92C

We used previously described methods^19^^,^^48^ to screen pooled moth legs for the T92C mutation, as detailed below.

#### DNA preparation from pooled legs

For each sample of moths from a given site and year, we extracted DNA with the phenol/chloroform method^19^ from a pool containing one hind leg from each moth. DNA was quantified with an OD-1000+ Spectrophotometer (OneDrop, Nanjing, China).

#### PCR amplification with tagged primers

To mark the amplicons from each site, we used a specific 6-bp tag for each site on the 5 prime end of the forward primer KI-F and the reverse primer KI-R.^19^ The amplicons were 216 bp (204 bp of the genomic DNA fragment and 12 bp from the two 6-bp tags). With specific tagged primers, specific amplicons can be determined for each site. This also reduces errors from chimeras in PCR amplifications. The 25-μl PCR mixture contained 0.5 μl of LA Taq DNA polymerase (TaKaRa, Dalian, China), 2.5 μl of 10× LA PCR buffer, 1 μM of each primer, 150 μM of each deoxynucleoside triphosphate, 2 mM of Mg^2+^, and 200 ng of DNA template. The PCR amplification conditions included denaturation at 94°C for 3 min, followed by 32 cycles (94°C for 30 s, 60°C for 30s, and 72°C for 30s) and a final extension at 72°C for 10 min. PCR products were separated by 1% agarose gels, and target DNA fragments were purified with AxyPrep DNA Gel Extraction Kit (Axygen Biosciences, CA).

#### Next-generation sequencing (NGS) of the pooled amplicons

The tagged purified PCR products for each year were pooled for NGS. The quantity of DNA from each site added to the pool was proportional to the number of legs from each site. The libraries were prepared using TruSeq DNA PCR-Free Kits (Illumina, CA). The pooled amplicons were Illumina sequenced at BIOZERON Biotechnology Ltd. (Shanghai, China). The Illumina platform was Novaseq 6000 with four lanes. Each sample was sequenced using paired ends and read length of 150 bp.

#### DNA reads sorted according to tags

We checked the quality of raw data using FastQCsoftware (http://www.bioinformatics.babraham.ac.uk/projects/fastqc). The raw data were trimmed using FASTX_Clipper (http://hannonlab.cshl.edu/fastx_toolkit/) with parameters ‘-a AGATCGGAAGAGC -Q 33’ and quality filtered using FASTQ_Quality_Filter (http://hannonlab.cshl.edu/fastx_toolkit/index.html) with parameters ‘-q 20 -p 80 -Q 33’. Next, the sequences were sorted by their barcodes using *Seqtk_demultiplex* software with parameter ‘-l 6’ (https://github.com/jameslz/fastx-utils). The sorted pair-end data were merged using *PANDAseq* with parameters ‘-F -o 50 -T 45 -L 300’^46^ and mapped to the reference using the BWA MEM algorithm with parameter ‘-M’.^47^ Target sequences were extracted with Python Script-1 from Guan et al.^48^ and analyzed using FASTX_Collapser (http://hannonlab.cshl.edu/fastx_toolkit/). To remove potential sequencing errors, we filtered the data as described previously^19^ using Python Script-2 from Guan et al.^48^ This filtering removed sequences if their frequency was less than 1/(2n), where n is the number of legs in the pool, which represents the theoretical minimal allele frequency for the sample.^19^ For the new data reported here for 2017–2020, values for the mean annual frequency of the T92C mutation were virtually identical with and without filtering (correlation: *r* = 1.00, df = 2, p < 0.001). The mean frequency of the T92C mutation for each year was nearly identical without weighting and with weighting by the square root of the number of individual legs from each site (*r* = 1.00, df = 9, p < 0.0001). For simplicity, we report the mean frequency without weighting.

#### Sequencing of individual resistant F_1_ moths to detect T92C

We used previously described methods^19^ to determine the T92C genotype (Table S3) of F_1_ individuals that were determined to be phenotypically resistant to Cry1Ac in bioassays. For each resistant F_1_ moth, we prepared genomic DNA from the whole abdomen with the phenol/chloroform method as described above. We used specific primers (KI-F and KI-R)^19^ to amplify a genomic DNA fragment of 204 bp, which contains T92C. The 25 μl PCR mixture contained 0.5 μl of LA Taq DNA polymerase (TaKaRa, Dalian, China), 2.5 μl of 10× LA PCR buffer, 1 μM of each primer, 150 μM of each deoxynucleoside triphosphate, 2 mM of Mg^2+^, 200 ng of DNA template. The PCR amplification was performed as denaturation at 94°C for 3 min, followed by 32 cycles (94°C for 30 s, 60°C for 30s, and 72°C for 30s) and a final extension at 72°C for 10 min. PCR products were purified and directly Sanger sequenced using the forward primer KI-F^19^ with an ABI 3730 by TSINGKE (Beijing, China).

#### Computer simulations

To assess the potential effects of dominance on evolution of *H. armigera* resistance to Bt cotton in northern China, we used a previously described deterministic population genetic model of *H. armigera* resistance to Bt cotton in northern China.^19^ We based modeling assumptions primarily on empirical data for *H. armigera* in northern China. Table S5 summarizes the parameter values we examined.

We chose a simple, previously described population genetic model^19^ for several reasons: to enable incorporation of realistic biological parameters for *H. armigera* in northern China, to examine expected outcomes of different assumptions about refuges and fitness cost using the same basic model, and to make the modeling results readily verifiable by readers. As reported previously, the results from this model match precisely with the results of Gould^14^ and are similar to results from models with much greater complexity, such as the results of Gustafson et al.^50^ Furthermore, predictions from this model corresponded well with the observed trajectory of *H. armigera* resistance to Cry1Ac in northern China from 2010 to 2016.^19^

We simulated a single locus (*HaTSPAN1*) with the mutant T92C allele (*r*) conferring resistance to Cry1Ac and a wild-type allele (*s*) conferring susceptibility. This ignores resistance alleles at other loci, which could overestimate the selective advantage of T92C and its rate of increase. We estimated the fitness of each of three genotypes (*ss, rs,* and *rr*) on Bt and non-Bt cotton based on empirical data with the respective plants for the field-derived resistant strain from northern China AY2 (*rr*), the susceptible strain SCD (*ss*), and their F_1_ progeny (*rs*)*.*^19^ For each year, we simulated three generations, corresponding to the three generations that *H. armigera* feeds on cotton in northern China.^26^^,^^51^

We modeled 2006 to 2020 to enable direct comparisons between the predicted frequency of T92C from the simulations and the observed frequency based on DNA screening for T92C in field populations from northern China from 2006 to 2020. To make robust comparisons between predicted and observed outcomes, we used the means for northern China as parameters in the model and for the observed frequency of T92C in field populations. We also conducted sensitivity analyses to assess the potential effects on resistance evolution of variation in the abundance of refuges and fitness cost of resistance (Table S5). We examined effects of *h* = 0.79 (dominant) as empirically observed. For the recessive fitness cost, we used the empirically observed value of 0.36, and hypothetical values of 0.18 and 0.54.

We assumed that mating occurred at random among adults of different genotypes. Because of the close proximity of small plantings of Bt cotton and non-Bt host plants and the extensive dispersal and gene flow of *H. armigera* in northern China, we also assumed that mating occurred at random among adults emerging from all host plants that overlapped substantially in time.^15^ Substantial gene flow occurs among populations of *H. armigera* in Africa, Asia, and Australia.^52^ Thus, in addition to the similar conditions among the provinces of northern China, we expect extensive gene flow in northern China to reduce differences in resistance among provinces in this region.^15^ Also, a previous analysis found no significant variation in resistance of *H. armigera* to Cry1Ac among provinces in northern China.^15^ We used data on the abundance of Bt cotton, non-Bt cotton^22^ and other *H. armigera* host plants in northern China and production of *H. armigera* adults on unsprayed non-Bt cotton, corn, peanut, and soybean in field cages^15^ to estimate the effective refuge percentage (Table S4).

Based on data for insecticide treatments from 2007 to 2019 in the six provinces of northern China we studied, the mean number of treatments targeting *H. armigera* on the non-Bt host plants that accounted for the majority of the refuge area (maize, peanut, and soybean) was 0.27 per ha per year. Thus, on average in a given year, 73% of the ha of these crops were not treated with insecticides targeting *H. armigera* and 27% received one treatment targeting *H. armigera.* With this low level of treatment, we expect that insecticides had minimal effects on *H. armigera* populations on non-Bt host plants.

Moreover, relative to mean yearly sprays per ha on non-Bt host plants, treatments targeting *H. armigera* were 7.4 times higher on cotton (Table S6, p < 0.0001) and treatments against all pests were 4.1 times higher on cotton (Table S7, p < 0.0001). Because cotton was predominantly Bt cotton (Table S4), the higher number of treatments for cotton than for non-cotton refuge crops suggests that insecticide treatments probably reduced the abundance of *H. armigera* in Bt cotton more than in the non-cotton refuge crops. However, to avoid overestimating the effective refuge percentage, we did not adjust it for insecticide treatments. Because this approach might underestimate the abundance of *H. armigera* in refuges relative to Bt cotton, our conclusion that the non-Bt crop refuges were sufficient to account for the observed delay in resistance is conservative.

### Quantification and statistical analysis

From sequencing of pooled moths, we used the output from FASTX_Collapser (http://hannonlab.cshl.edu/fastx_toolkit/) to obtain the raw number of counts for the T92C mutation and for all alleles. After trimming, quality control checking, demultiplexing, and filtering as described above, we calculated the frequency of the T92C mutation for each site in each year as the number of sequences that had this mutation divided by the total number of sequences (Table S1). We used the bootstrap method with 1000 repetitions to estimate the 95% confidence interval for each of the estimates of the annual mean frequency of the T92C mutation for the pooled data from field populations. From sequencing of individual F_1_ moths that were resistant based on their survival in bioassays with Cry1Ac, we determined the genotype of each moth for T92C and used the individual genotypes to calculate the frequency of T92C in the resistant F_1_ moths (Table S3). We calculated the 95% confidence interval for the T92C frequency based on resistant F_1_ moths using the Wilson procedure with continuity correction (http://vassarstats.net/prop1.html). We used linear regression of log-transformed data for field populations to evaluate the association between year and both the mean yearly T92C frequency and the percentage of resistant individuals in bioassays. We also used linear regression to evaluate the association between year and the yearly T92C frequency for F_1_ individuals determined to be resistant based on bioassays. We used correlation analysis to evaluate the association between the two independent metrics used for monitoring resistance in field populations: T92C frequency and percentage of resistant individuals in bioassays. Measurements were taken from distinct samples and all tests were two-sided.

## Data Availability

• The Illumina data of amplicon sequencing for detecting the frequency of the T92C mutation in field cotton bollworm were deposited with BioProject No. PRJNA919153. • This paper does not report original code. • Any additional information required to reanalyze the data reported in this paper is available from the lead contact upon request.

## Supplemental information

Ducument S1. Figures S1–S3 and Tables S1–S7
