# Supplementary material for: Refuges of conventional host plants counter dominant resistance of cotton bollworm to transgenic Bt cotton
Source: iScience. 2023 Apr 26;26(5):106768. doi: 10.1016/j.isci.2023.106768 (PMC10196555; doi:10.1016/j.isci.2023.106768)
Supplement: Ducument S1. Figures S1–S3 and Tables S1–S7 [file mmc1.pdf]

iScience, Volume *26*

Supplemental information

Refuges of conventional host plants counter dominant resistance of cotton bollworm to transgenic Bt cotton

Fang Guan, Xiaoguang Dai, Bofeng Hou, Shuwen Wu, Yihua Yang, Yanhui Lu, Kongming Wu, Bruce E. Tabashnik, and Yidong Wu

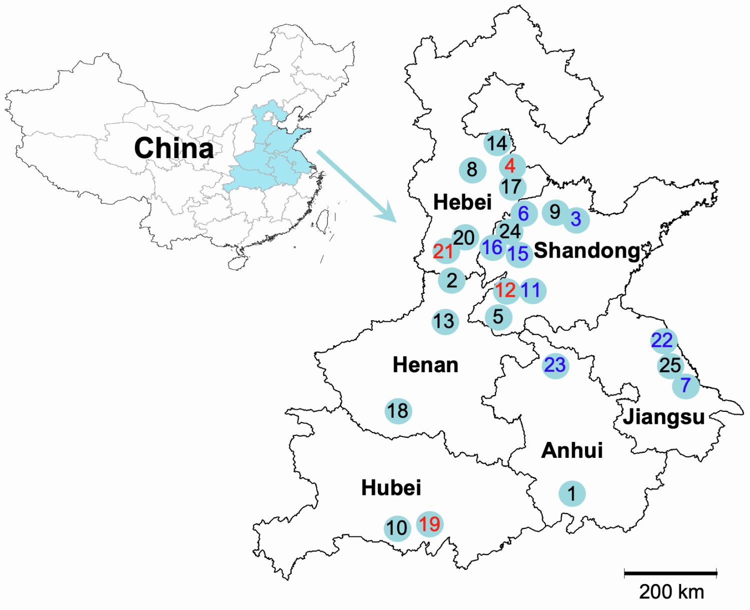

Code	Sampling site	Province
1	Anqing (AQ)	Anhui
2	Anyang (AY)	Henan
3	Binzhou (BZ)	Shandong
4	Cangxian (CG)	Hebei
5	Caoxian (CX)	Shandong
6	Dezhou (DZ)	Shandong
7	Dongtai (DT)	Jiangsu
8	Gaoyang (GY)	Hebei
9	Huimin (HM)	Shandong
10	Jingzhou (JZ)	Hubei
11	Jining (JN)	Shandong
12	Juye (JY)	Shandong
13	Kaifeng (KF)	Henan
14	Langfang (LF)	Hebei
15	Liaocheng (LC)	Shandong
16	Linqing (LQ)	Shandong
17	Nanpi (NP)	Hebei
18	Nanyang (NY)	Henan
19	Qianjiang (QJ)	Hubei
20	Qiuxian (QX)	Hebei
21	Quzhou (QZ)	Hebei
22	Sheyang (SY)	Jiangsu
23	Suzhou (SZ)	Anhui
24	Xiajin (XJ)	Shandong
25	Yancheng (YC)	Jiangsu

Fig. S1. Sampling sites for monitoring resistance of *H. armigera* in six provinces of northern China, Related to STAR Methods. The color of the 25 site names in the key above indicates the monitoring methods used. Black: both DNA screening and bioassays (13 sites). Blue: only DNA screening (8 sites). Red: only bioassays (4 sites) (Tables S1-S3).

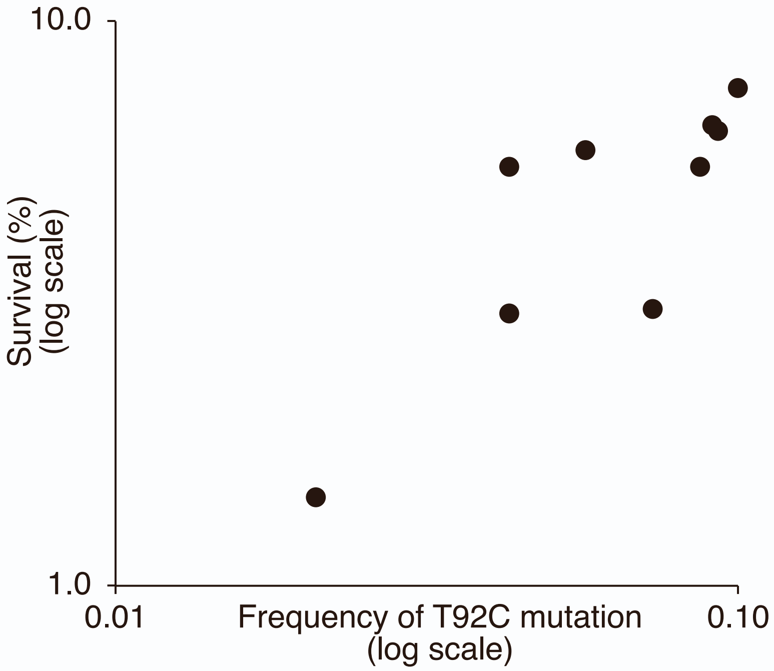

Fig. S2. Correlation between the frequency of the T92C mutation and larval survival in bioassays at the diagnostic concentration of Cry1Ac: 2010 and 2012-2020, Related to Figure 1. Each point represents data from one year. Results from correlation analysis with log transformation of both parameters: r = 0.84, df = 8, P = 0.002.

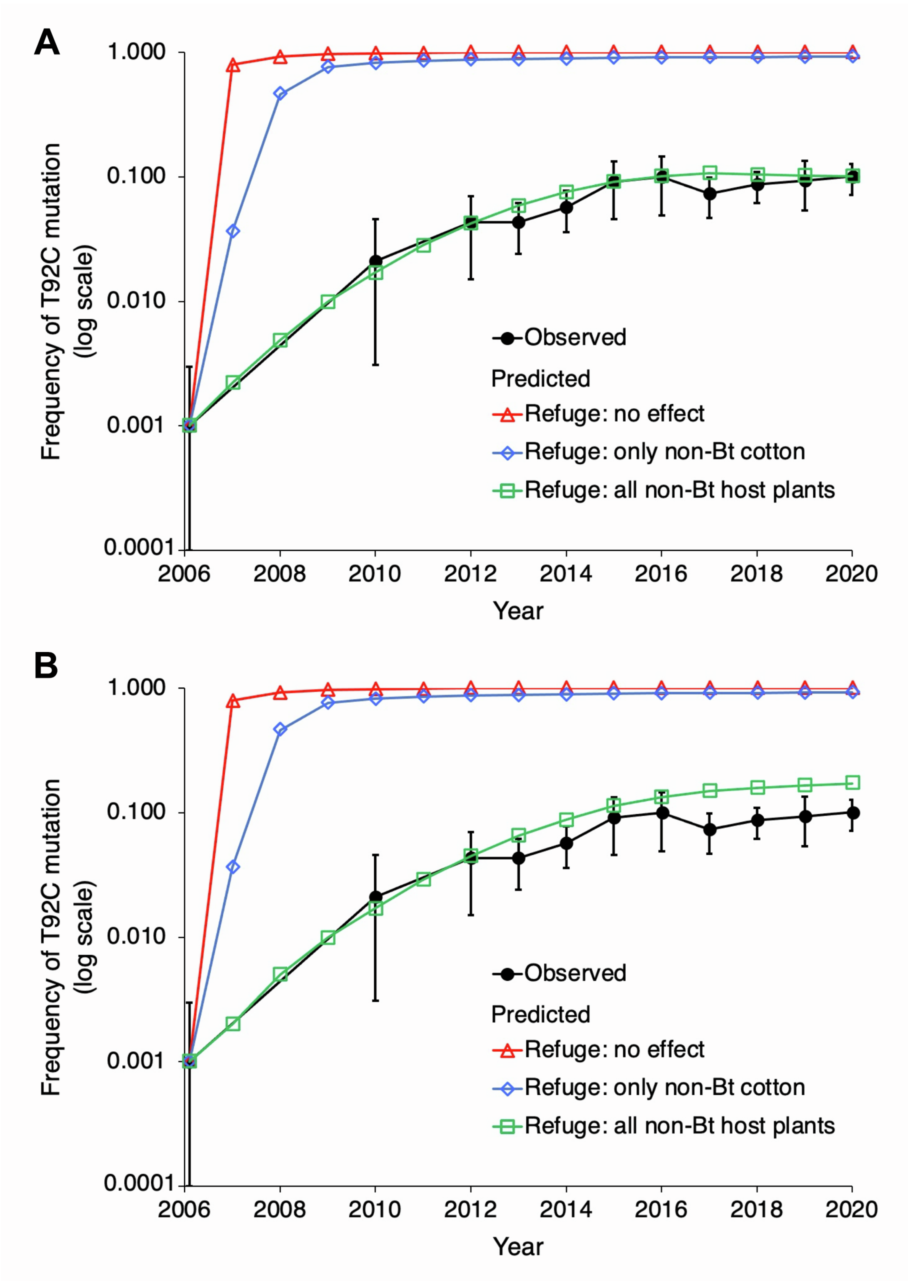

Fig. S3. Sensitivity analysis: Effects of variation in fitness cost and effective refuge percentage on the correspondence between predicted and observed frequency of the T92C mutation in *H. armigera* in northern China, Related to Figure 4. The observed values are means (with 95% confidence interval, CI) based on DNA screening. The predicted values are from simulations of a population genetic model using the parameters listed in Table S5. In all simulations, the observed frequency for 2006 of 0.001 (95% CI: 0.0 to 0.003) was used as the initial frequency (i.e., for 2006); and dominant resistance (h = 0.79) as determined empirically (Jin *et al.*, 2018). As empirically determined, fitness on non-Bt cotton was 1 for susceptible homozygotes (*ss*) and heterozygotes (*rs*). Based on empirical data, the standard fitness of *rr* was 0.64 on non-Bt cotton (Jin

et al., 2018), which yields a recessive fitness cost of 0.36 (1 – 0.64). We tested three different assumptions about refuges: i) refuges had no effect, ii) only refuges of non-Bt cotton plants delayed resistance, and iii) non-Bt cotton and other non-Bt host plants contribute to the effective refuge percentage delayed resistance. The recessive fitness cost was 0.54 for (**A**) and 0.18 for (**B**). With all three values of fitness cost examined (including 0.36 in Fig. 4), the best fit between the predicted and observed frequency of T92C occurred with assumption iii) non-Bt cotton and other non-Bt host plants delayed resistance.

Table S1. Frequency of the T92C mutation in pools of field-captured *H. armigera* moths from northern China, Related to STAR Methods.

							T92C mutation frequency		
Year	Sampling site	Province	n[a]	Total counts	Coverage	T92C counts	Per pool	Yearly mean (95% CI)[b]	Total moths per year
2006	Dongtai (DT)	Jiangsu	115	3,640	32	0	0.0000	0.001	770
2006	Sheyang (SY)	Jiangsu	36	130	4	0	0.0000	(0.000 - 0.003)	
2006	Binzhou (BZ)	Shandong	140	3,639	26	0	0.0000		
2006	Dezhou (DZ)	Shandong	59	51,798	878	0	0.0000		
2006	Huimin (HM)	Shandong	120	5,261	44	0	0.0000		
2006	Jining (JN)	Shandong	90	481	5	0	0.0000		
2006	Liaocheng (LC)	Shandong	140	1,712	12	0	0.0000		
2006	Linqing (LQ)	Shandong	70	45,549	651	416	0.0091		
2010	Gaoyang (GY)	Hebei	27	4,887	181	92	0.0188	0.021	469
2010	Langfang (LF)	Hebei	25	480	19	10	0.0208	(0.003 - 0.046)	
2010	Qiuxian (QX)	Hebei	80	33,082	414	2,810	0.0849		
2010	Anyang (AY)	Henan	250	6,355	25	0	0.0000		
2010	Kaifeng (KF)	Henan	65	65,584	1,009	0	0.0000		
2010	Nanyang (NY)	Henan	22	27,466	1,248	0	0.0000		
2012	Anqing (AQ)	Anhui	105	86,867	827	2,994	0.0345	0.043	501
2012	Gaoyang (GY)	Hebei	48	32,427	676	3,325	0.1025	(0.015 - 0.070)	
2012	Nanpi (NP)	Hebei	61	3,789	62	284	0.0750		
2012	Yancheng (YC)	Jiangsu	195	85,999	441	1,054	0.0123		
2012	Huimin (HM)	Shandong	50	60,124	1,202	1,901	0.0316		
2012	Xiajin (XJ)	Shandong	42	42,961	1,023	0	0.0000		
2013	Anqing (AQ)	Anhui	19	161,126	8,480	7,953	0.0494	0.043	962
2013	Nanpi (NP)	Hebei	57	870	15	29	0.0333	(0.024 - 0.062)	
2013	Qiuxian (QX)	Hebei	183	20,546	112	1,668	0.0812		
2013	Anyang (AY)	Henan	35	585	17	46	0.0786		
2013	Kaifeng (KF)	Henan	165	102,779	623	3,353	0.0326		
2013	Jingzhou (JZ)	Hubei	80	1,489	19	0	0.0000		
2013	Yancheng (YC)	Jiangsu	250	1,064	4	14	0.0132		
2013	Huimin (HM)	Shandong	55	16,377	298	1,266	0.0773		
2013	Xiajin (XJ)	Shandong	118	1,700	14	38	0.0224		
2014	Anqing (AQ)	Anhui	65	201,406	3,099	4,677	0.0232	0.057	1568
2014	Suzhou (SZ)	Anhui	190	91,608	482	0	0.0000	(0.036 - 0.078)	
2014	Gaoyang (GY)	Hebei	31	2,416	78	222	0.0919		
2014	Langfang (LF)	Hebei	180	1,478	8	139	0.0940		
2014	Nanpi (NP)	Hebei	131	7,044	54	507	0.0720		
2014	Qiuxian (QX)	Hebei	237	35,884	151	4,522	0.1260		
2014	Anyang (AY)	Henan	228	1,213	5	79	0.0651		
2014	Kaifeng (KF)	Henan	156	65,513	420	1,340	0.0205		
2014	Jingzhou (JZ)	Hubei	18	200,070	11,115	0	0.0000		
2014	Yancheng (YC)	Jiangsu	42	40,518	965	503	0.0124		
2014	Caoxian (CX)	Shandong	80	7,750	97	720	0.0929		
2014	Huimin (HM)	Shandong	96	48,725	508	3,376	0.0693		
2014	Xiajin (XJ)	Shandong	114	590	5	40	0.0678		
2015	Anqing (AQ)	Anhui	102	1,061	10	0	0.0000	0.091	1071
2015	Gaoyang (GY)	Hebei	88	7,341	83	1,047	0.1426	(0.046 - 0.133)	
2015	Langfang (LF)	Hebei	227	726	3	135	0.1860		
2015	Nanpi (NP)	Hebei	105	70,207	669	10,358	0.1475		
2015	Qiuxian (QX)	Hebei	122	66,491	545	11,039	0.1660		
2015	Kaifeng (KF)	Henan	92	91,869	999	2,357	0.0257		
2015	Yancheng (YC)	Jiangsu	120	4,066	34	49	0.0121		
2015	Huimin (HM)	Shandong	90	136,709	1,519	5,303	0.0388		
2015	Xiajin (XJ)	Shandong	125	139,960	1,120	13,660	0.0976		
2016	Anqing (AQ)	Anhui	89	617	7	0	0.0000	0.100	655
2016	Gaoyang (GY)	Hebei	33	308	9	15	0.0487	(0.049 - 0.145)	
2016	Langfang (LF)	Hebei	100	1,352	14	204	0.1509		
2016	Nanpi (NP)	Hebei	61	47,368	777	6,907	0.1458		
2016	Qiuxian (QX)	Hebei	105	157,335	1,498	24,268	0.1542		
2016	Anyang (AY)	Henan	92	506	6	11	0.0217		
2016	Huimin (HM)	Shandong	74	352	5	30	0.0852		
2016	Xiajin (XJ)	Shandong	101	457	5	88	0.1926		
2017	Langfang(LF)	Hebei	97	89366	921	5250	0.0587	0.073	658
2017	Nanpi(NP)	Hebei	92	121054	1316	12178	0.1006	(0.047-0.099)	
2017	Qiuxian(QX)	Hebei	79	131628	1666	13776	0.1047		
2017	Anyang(AY)	Henan	98	1202482	12270	51250	0.0426		
2017	Kaifeng(KF)	Henan	69	51018	739	3262	0.0639		
2017	Jingzhou(JZ)	Hubei	130	329252	2533	6544	0.0199		
2017	Xiajin(XJ)	Shandong	93	116468	1252	14134	0.1214		
2018	Anqing(AQ)	Anhui	100	264920	2649	7176	0.0271	0.087	1,350
2018	Gaoyang(GY)	Hebei	50	326738	6535	42176	0.1291	(0.062-0.110)	
2018	Langfang(LF)	Hebei	250	897558	3590	84640	0.0943		
2018	Nanpi(NP)	Hebei	150	621640	4144	74688	0.1201		
2018	Qiuxian(QX)	Hebei	200	994176	4971	136686	0.1375		
2018	Anyang(AY)	Henan	200	1258378	6292	94588	0.0752		
2018	Kaifeng(KF)	Henan	200	1144140	5721	55802	0.0488		
2018	Jingzhou(JZ)	Hubei	50	213572	4271	9092	0.0426		
2018	Huimin(HM)	Shandong	150	351692	2345	36606	0.1041		
2019	Gaoyang(GY)	Hebei	50	271472	5429	29962	0.1104	0.093	900
2019	Nanpi(NP)	Hebei	50	274626	5493	29508	0.1074	(0.054-0.134)	
2019	Qiuxian(QX)	Hebei	200	1444766	7224	284942	0.1972		
2019	Anyang(AY)	Henan	200	1440314	7202	47748	0.0332		
2019	Kaifeng(KF)	Henan	200	1668550	8343	75340	0.0452		
2019	Yancheng(YC)	Jiangsu	100	790166	7902	31236	0.0395		
2019	Huimin(HM)	Shandong	50	195280	3906	32136	0.1646		
2019	Xiajin(XJ)	Shandong	50	153690	3074	6812	0.0443		
2020	Gaoyang(GY)	Hebei	200	714724	3574	76714	0.1073	0.101	2,050
2020	Langfang(LF)	Hebei	200	727806	3639	117148	0.1610	(0.072-0.127)	
2020	Nanpi(NP)	Hebei	150	747952	4986	106606	0.1425		
2020	Qiuxian(QX)	Hebei	300	1867746	6226	280590	0.1502		
2020	Anyang(AY)	Henan	300	1761338	5871	146160	0.0830		
2020	Kaifeng(KF)	Henan	300	1145436	3818	67976	0.0593		
2020	Yancheng(YC)	Jiangsu	50	160504	3210	2498	0.0156		
2020	Huimin(HM)	Shandong	250	1231898	4928	116146	0.0943		
2020	Xiajin(XJ)	Shandong	300	1304422	4348	120584	0.0924		

[a] Moths tested per pool, total of 10,954 moths tested in 92 pools, mean = 119 moths per pool (SE = 6)

[b] 95% confidence interval calculated by the bootstrap method with 1,000 repetitions; http://www.lock5stat.com/StatKey/bootstrap_1_quant/bootstrap_1_quant.html

Data for 2006-2016 were reported previously (Jin *et al.*, 2018).

Table S2. Survival at a diagnostic concentration of Cry1Ac for F_1 progeny of *H. armigera* from northern China[a], Related to Figure 1 and STAR Methods.

Site	Province	Survival (%) of F_1 larvae at diagnostic concentration (1 µg Cry1Ac per cm^2 diet)										
		2010	2011	2012	2013	2014	2015	2016	2017	2018	2019	2020
Anqing (Aq)	Anhui			3.03	2.20	1.49	1.50	0.20		0.00	0.00	0.00
Anyang (Ay)	Henan	2.64	2.31	1.88	3.50	3.08	8.23	4.86	2.88	6.45	12.42	11.51
Cangxian (Cg)	Hebei		2.08			5.36						
Caoxian (Co)	Hebei		1.13									
Gaoyang (Gy)	Hebei	0.00	0.21	2.42	9.92	5.06	8.33	16.80	2.75	13.99	3.75	16.67
Huimin (Hm)	Shandong	0.79	3.61	6.05	3.87	12.90	7.44	8.80	6.35	4.66	8.67	18.39
Jingzhou (Jz)	Hubei			0.57	0.30	0.40	0.30	0.20	0.47	0.83	0.00	0.00
Juye (Jy)	Shandong	0.00										
Kaifeng (Kf)	Henan	0.00	0.20		11.01	4.07	4.76	7.60	0.30	2.78	6.75	8.96
Langfang(LF)	Hebei	1.60	0.97	1.59	11.31	12.60	10.91	14.68	5.81	8.93	7.92	9.74
Nanpi (Np)	Hebei	2.22	2.69	8.04	3.47	6.25	10.02	11.80	5.68	9.03	13.25	7.78
Nanyang (Ny)	Henan	1.70										
Qianjiang (Qj)	Hubei	0.00	0.00									
Qiuxian (Qx)	Hebei	0.23	1.43		2.88	9.13	10.02	6.50	4.01	7.34	8.83	13.03
Quzhou (Qz)	Hebei	0.00	0.74									
Xiajin (Xj)	Shandong	2.48	2.33	2.48	6.15	9.72	8.53	10.42	0.48	6.65	8.44	16.04
Yancheng (Yc)	Jiangsu	0.40	0.95	1.19	5.90	0.60	1.80	1.59	2.08	0.00	0.00	0.00
Mean	survival	0.93	1.4	3.0	5.5	5.9	6.5	7.6	3.1	5.5	6.4	9.3
	Sites	13	13	9	11	12	11	11	10	11	11	11
	n[b]	11,064	13,968	8,592	11,088	12,096	11,088	11,088	10,560	11,160	13,224	15,480

[a] Data from 2010 to 2013 were reported previously (Jin *et al.*, 2015).

[b] Number of F_1 larvae tested at the diagnostic concentration

Table S3. Frequency of the T92C mutation in survivors of exposure to the diagnostic concentration of Cry1Ac from northern China in 2011-2020, Related to Figure 2 and STAR Methods.

Year	n[a]	Homozygous mutant (92C/92C)	Heterozygous mutant (92T/92C)	Homozygous wild type (92T/92T)	T92C allele frequency[b]
2011[c]	18	0	8	10	0.222
2012	25	0	13	12	0.260
2013	142	1	90	51	0.324
2014	219	10	139	70	0.363
2015	64	5	51	8	0.477
2016	108	11	65	32	0.403
2017	72	3	51	18	0.396
2018	135	12	104	19	0.474
2019	227	57	125	45	0.526
2020	360	68	223	69	0.499

[a] Number of survivors of exposure to the diagnostic concentration of Cry1Ac screened individually for the T92C mutation. The survivors were from the F_1 offspring of field-collected insects (see Methods).

[b] Calculated as (number of heterozygous mutants + 2 $\times$ number of homozygous mutants)/(2 $\times$ n)

[c] Data from 2011 to 2016 were reported previously (Jin *et al.*, 2018).

Table S4. Area of *H. armigera* host plants and effective refuge % in northern China, 2007-2019, Related to Figure 3.

Year	Host plant	Area planted[a] (1000 ha)	% of total host plant area	RRC[b]	Generation 2[c] Effective refuge (1000 ha)	Generation 2[c] Effective refuge %	Generations 3 and 4[c] Effective refuge (1000 ha)	Generations 3 and 4[c] Effective refuge %	Mean effective refuge (%)[d]
2007	Cotton	3,499	13.38	0.14	490	8	490	7	
2007	Maize	10,039	38.40	0, 0.10	0	0	1,004	14	
2007	Peanut	2,537	9.71	0.78	1,979	32	1,979	27	
2007	Legumes	2,493	9.54	0.30	748	12	748	10	
2007	Other	7,574	28.97	0.00	0	0	0	0	
2007	Total	26,142	100		3,217	51.67	4,221	58.38	56
2008	Cotton	3,420	12.88	0.12	410	7	410	6	
2008	Maize	10,115	38.11	0, 0.10	0	0	1,011	14	
2008	Peanut	2,640	9.95	0.78	2,060	33	2,060	28	
2008	Legumes	2,596	9.78	0.30	779	12	779	11	
2008	Other	7,771	29.28	0.00	0	0	0	0	
2008	Total	26,542	100		3,249	51.91	4,260	58.60	56
2009	Cotton	3,023	11.43	0.12	363	6	363	5	
2009	Maize	10,406	39.33	0, 0.10	0	0	1,041	15	
2009	Peanut	2,611	9.87	0.78	2,037	35	2,037	30	
2009	Legumes	2,504	9.46	0.30	751	13	751	11	
2009	Other	7,916	29.91	0.00	0	0	0	0	
2009	Total	26,460	100		3,151	54.22	4,192	61.17	59
2010	Cotton	2,875	10.38	0.23	661	12	661	10	
2010	Maize	10,606	38.30	0, 0.10	0	0	1,061	16	
2010	Peanut	2,649	9.57	0.78	2,066	36	2,066	31	
2010	Legumes	2,422	8.75	0.30	727	13	727	11	
2010	Other	9,141	33.00	0.00	0	0	0	0	
2010	Total	27,694	100		3,454	60.94	4,515	67.10	65
2011	Cotton	2,860	10.23	0.25	715	13	715	11	
2011	Maize	10,839	38.78	0, 0.10	0	0	1,084	16	
2011	Peanut	2,649	9.48	0.78	2,066	37	2,066	31	
2011	Legumes	2,344	8.39	0.30	703	12	703	10	
2011	Other	9,258	33.12	0.00	0	0	0	0	
2011	Total	27,950	100		3,484	61.90	4,568	68.05	66
2012	Cotton	2,473	8.86	0.25	618	12	618	10	
2012	Maize	11,002	39.39	0, 0.10	0	0	1,100	17	
2012	Peanut	2,672	9.57	0.78	2,084	40	2,084	33	
2012	Legumes	2,314	8.29	0.30	694	13	694	11	
2012	Other	9,469	33.91	0	0	0	0	0	
2012	Total	27,930	100		3,397	64.68	4,497	70.80	69
2013	Cotton	2,199	7.87	0.27	594	12	594	10	
2013	Maize	11,217	40.14	0, 0.10	0	0	1,122	18	
2013	Peanut	2,654	9.5	0.78	2,070	42	2,070	34	
2013	Legumes	2,247	8.04	0.30	674	14	674	11	
2013	Other	9,629	34.46	0.00	0	0	0	0	
2013	Total	27,946	100		3,338	67.53	4,460	73.53	72
2014	Cotton	1,899	6.78	0.26	494	11	494	9	
2014	Maize	11,511	41.12	0, 0.10	0	0	1,151	20	
2014	Peanut	2,645	9.45	0.78	2,063	45	2,063	36	
2014	Legumes	2,200	7.86	0.30	660	14	660	11	
2014	Other	9,741	34.78	0.00	0	0	0	0	
2014	Total	27,996	100		3,217	69.6	4,368	75.66	74
2015	Cotton	1,585	5.65	0.26	412	10	412	8	
2015	Maize	11,788	42.04	0, 0.10	0	0	1,179	22	
2015	Peanut	2,639	9.41	0.78	2,058	48	2,058	38	
2015	Legumes	2,067	7.37	0.30	620	15	620	11	
2015	Other	9,960	35.52	0.00	0	0	0	0	
2015	Total	28,039	100		3,091	72.49	4,269	78.45	76
2016	Cotton	1,302	4.68	0.26	339	8	339	7	
2016	Maize	11,697	42.04	0, 0.10	0	0	1,170	22	
2016	Peanut	2,693	9.68	0.78	2,101	52	2,101	40	
2016	Legumes	2,101	7.55	0.30	630	16	630	12	
2016	Other	10,029	36.04	0.00	0	0	0	0	
2016	Total	27,822	100		3,069	76.11	4,239	81.48	80
2017	Cotton	985	3.56	0.26	256	7	256	5	
2017	Maize	14,041	50.77	0, 0.10	0	0	1,404	28	
2017	Peanut	2,586	9.35	0.78	2017	57	2,017	41	
2017	Legumes	1,752	6.33	0.30	526	15	526	11	
2017	Other	8,294	29.99	0.00	0	0	0	0	
2017	Total	27,657	100		2,798	79.34	4,202	85.22	83
2018	Cotton	693	6.78	0.26	180	5	180	4	
2018	Maize	13,727	41.12	0, 0.10	0	0	1,373	29	
2018	Peanut	2,632	9.45	0.78	2,053	62	2,053	44	
2018	Legumes	1,890	7.86	0.30	567	17	567	12	
2018	Other	8,456	34.78	0.00	0	0	0	0	
2018	Total	27,397	100		2,800	84.53	4,172	89.06	88
2019	Cotton	642	5.65	0.26	167	5	167	4	
2019	Maize	13,484	42.04	0, 0.10	0	0	1,348	29	
2019	Peanut	2,629	9.41	0.78	2,051	63	2,051	44	
2019	Legumes	1,920	7.37	0.30	576	18	576	12	
2019	Other	8,557	35.52	0.00	0	0	0	0	
2019	Total	27,232	100		2,794	85.47	4,142	89.71	88

Notes for Table S4

[a] Data from the Chinese Ministry of Agriculture for the six provinces we studied: Anhui, Hebei, Henan, Hubei, Jiangsu, and Shandong.

[b] We calculated effective refuge ha for each host plant as ha of the host plant X RRC, where RRC is the relative refuge contribution per ha. We calculated RRC as the number of *H. armigera* moths produced per ha relative to non-Bt cotton based on data from field cage experiments with corn (maize), peanut and soy (Jin *et al.*, 2015).

We calculated RRC for cotton as non-Bt cotton ha divided by total cotton ha. Values for RRC of cotton were 0.14 for 2007, 0.12 for 2008 and 2009, 0.23 for 2010, 0.25 for 2011 and 2012, 0.27 for 2013, and 0.26 for 2014-2019. For 2007 to 2015, we based RRC for cotton on previously reported data for the percentage of cotton ha planted with non-Bt cotton in the Yangtze River Valley of China (Wan *et al.*, 2017). This is a reasonable approximation because three of the six provinces in northern China that we analyzed here (Anhui, Hubei, and Jiangsu) were included in the previous study (Wan *et al.*, 2017) and we expect a similar percentage of non-Bt cotton in the other three provinces studied here. For 2016-2019, we used the percentage of non-Bt cotton from 2014-2015 (Wan *et al.*, 2017).

Because of the timing of planting, corn can contribute as a refuge during the third and fourth generations, but not the second generation of *H. armigera* (Wu *et al.*, 2002). Corn is planted at two different times (referred to as early and late) in northern China (Wu *et al.*, 2002). For simplicity, we assumed that half of the corn was planted early and contributed as a refuge during generation 3, and the other half was planted late and contributed as a refuge during generation 4. Therefore, to estimate the refuge contribution of corn relative to non-Bt cotton for generations 3 and 4, we divided the production of adults per ha of corn relative to non-Bt cotton (0.20) by two, which yields 0.10.

The RRC value we used for legumes (0.30) is based on the field cage data for soy. We used 0 as the RRC for Bt cotton and "other" host plants (melons, sesame, sorghum and vegetables, as listed by the Chinese Ministry of Agriculture), which may underestimate the total effective refuge percentage.

[c] In northern China, *H. armigera* feeds on wheat during its first generation, and cotton and other host plants during its second to fourth generations (Wu *et al.*, 2002).

[d] We calculated the mean effective refuge (%) for each year from 2007 to 2020 as the mean of the effective refuge (%) for the three generations *H. armigera* feeds on cotton (generations 2-4).

Data for 2007 to 2016 were reported previously (Jin *et al.*, 2018).

Table S5. Parameter values used in computer simulations, Related to STAR Methods.

Parameter	Values[a]	Source
Fitness		Jin *et al.*, 2015, 2018
Bt cotton[b]		
ss	0	
rs	0.390	
rr	0.495	
Non-Bt host plants		
ss	1	
rs	1	
rr	0.64[c], *0.46*[c], *0.88*[c]	
Initial frequency of T92C mutation (2006)	0.001	Jin *et al.*, 2018
Generations per year larvae feed on cotton	3	Wu and Guo, 2005
Refuge (%)[d]		Table S4
Refuges have no effect (hypothesis i)	*0* (for each year)	
Only non-Bt cotton (hypothesis ii)	*14, 12, 12, 23, 25, 25, 27, 26, 26, 26, 26, 26, 26, 26*	
Non-Bt cotton and other non-Bt host plants (hypothesis iii)	56, 56, 59, 65, 66, 69, 72, 74, 76, 80, 83, 86, 88, 88	

[a] Italics indicate hypothetical values examined in sensitivity analyses (Figs. 4 and S3)
[b] h = 0.79
[c] Fitness cost = 1 – fitness, so the values of the recessive fitness cost examined are 0.36 (standard), 0.54, and 0.12, respectively.
[d] Mean effective refuge percentage annually for *H. armigera* during the three generations its larvae fed on cotton each year from 2007 to 2020 (Table S4).

Table S6. Insecticide treatments in cotton and non-Bt crops targeting *H. armigera* in northern China (2007 - 2019), Related to STAR Methods.

	Insecticide treatments targeting *H. armigera* (mean per ha per year)				
Year	Maize	Peanut	Soybean	Non-cotton	Cotton
2007	0.21	0.30	0.18	0.23	2.13
2008	0.23	0.22	0.16	0.21	2.20
2009	0.24	0.26	0.24	0.25	2.25
2010	0.25	0.34	0.23	0.27	2.12
2011	0.15	0.36	0.12	0.21	2.11
2012	0.19	0.36	0.13	0.23	2.22
2013	0.26	0.47	0.23	0.32	2.19
2014	0.29	0.43	0.16	0.29	2.17
2015	0.28	0.39	0.17	0.28	1.98
2016	0.26	0.39	0.16	0.27	1.73
2017	0.31	0.47	0.21	0.33	1.80
2018	0.31	0.47	0.24	0.34	1.53
2019	0.33	0.45	0.25	0.34	1.45
Mean	0.25	0.38	0.19	0.27	1.99

We obtained the data on insecticide treatments from the Agricultural Technology Extension Service Center of the Ministry of Agriculture and Rural Affairs for the six provinces in northern China studied here (Anhui, Hebei, Henan, Hubei, Jiangsu, and Shandong). Non-cotton indicates the mean for maize, peanut, and soybean. The mean number of insecticide treatments per ha per year targeting *H. armigera* was higher for cotton (1.99) than for the mean of the three other crops (0.27) (Paired t-test, df = 12, t = 20.1, $P < 0.0001$).

Table S7. Insecticide treatments in cotton and non-Bt crops targeting all pests in northern China (2007 - 2019), Related to STAR Methods.

	Insecticide treatments targeting all pests (mean per ha per year)				
Year	Maize	Peanut	Soybean	Non-cotton	Cotton
2007	1.67	1.35	1.60	1.54	6.81
2008	1.61	1.31	1.47	1.46	7.84
2009	1.56	1.42	1.79	1.59	8.46
2010	1.64	1.57	1.78	1.66	7.27
2011	1.92	1.76	1.53	1.74	7.42
2012	1.91	1.90	1.57	1.79	7.70
2013	2.01	1.95	1.61	1.86	7.37
2014	2.07	1.79	1.38	1.74	7.65
2015	1.95	1.88	1.37	1.73	7.03
2016	1.80	1.71	1.33	1.61	6.48
2017	1.87	1.79	1.40	1.69	6.56
2018	2.39	3.15	1.74	2.43	7.89
2019	2.28	2.75	1.69	2.24	6.96
Mean	1.90	1.87	1.56	1.78	7.34

We obtained the data on insecticide treatments from the Agricultural Technology Extension Service Center of the Ministry of Agriculture and Rural Affairs for the six provinces in northern China studied here (Anhui, Hebei, Henan, Hubei, Jiangsu, and Shandong). Non-cotton indicates the mean for maize, peanut, and soybean. The mean number of insecticide treatments per ha per year targeting all pests was higher for cotton (7.34) than the mean for the three other crops (1.78) (Paired t-test, df = 12, t = 32.9, P < 0.0001).
